# Myeloid Sarcoma Involving the Testicular Vein

**DOI:** 10.4274/tjh.galenos.2020.2020.0436

**Published:** 2021-08-25

**Authors:** Nuh Filizoğlu, Salih Özgüven

**Affiliations:** 1 Marmara University Pendik Training and Research Hospital, Clinic of Nuclear Medicine, İstanbul, Turkey

**Keywords:** Myeloid sarcoma, Testicular vein, Leukemia

A 66-year-old man with a diagnosis of acute myeloid leukemia (AML) type M4 in 2017 who had undergone allogeneic hematopoietic stem cell transplantation in January 2018 presented with painless, nontender left hemiscrotal swelling. The patient underwent unilateral radical orchiectomy and histopathology revealed myeloperoxidase-, CD33-, and CD117-positive and CD34-negative infiltration of AML in the testis and local spread into the spermatic cord, rete testis, epididymis, tunica albuginea, and the surrounding soft tissue suggesting myeloid sarcoma (MS). F18-fluorodeoxyglucose positron emission tomography/computed tomography (FDG PET/CT) after left orchiectomy depicted moderate hypermetabolic metastatic retrocrural and paraaortic lymph nodes and multiple intense hypermetabolic foci along the course of the left testicular vein extending up to the left renal vein, suggesting testicular vein infiltration of MS (arrows in Figures 1A-1C). MS is a rare neoplasm of leukemic cells that infiltrates extramedullary soft tissue. Testicular involvement of MS is an uncommon entity, especially following hematopoietic stem cell transplantation, and invasion of MS into the spermatic cord and testicular vein is even rarer [1,2]. Although FDG PET/CT is well established for detecting, staging, and monitoring response to treatment in MS, this is an extremely rare condition of testicular involvement with invasion of the testicular vein not described before [3,4].

## Figures and Tables

**Figure 1:**
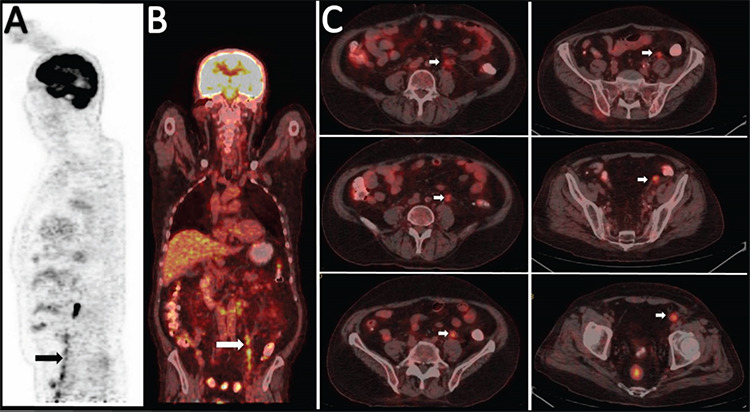
(A-C) F18-FDG PET/CT after left orchiectomy depicted moderate hypermetabolic metastatic retrocrural and paraaortic lymph nodes and multiple intense hypermetabolic foci along the course of the left testicular vein extending up to the left renal vein suggesting testicular vein infiltration of myeloid sarcoma (arrows). F18-FDG PET/CT: F18-fluorodeoxyglucose positron emission tomography/computed tomography.
